# Risk of metabolic abnormalities in osteoarthritis: a new perspective to understand its pathological mechanisms

**DOI:** 10.1038/s41413-023-00301-9

**Published:** 2023-12-06

**Authors:** Guizheng Wei, Ke Lu, Muhammad Umar, Zhenglin Zhu, William W. Lu, John R. Speakman, Yan Chen, Liping Tong, Di Chen

**Affiliations:** 1 Department of Bone and Joint Surgery, the First Affiliated Hospital of Guangxi Medical University, Nanning, 530021 China; 2 Research Center for Computer-aided Drug Discovery, Shenzhen Institute of Advanced Technology, Chinese Academy of Sciences, Shenzhen, 518055 China; 3 Faculty of Pharmaceutical Sciences, Shenzhen Institute of Advanced Technology, Chinese Academy of Sciences, Shenzhen, 518055 China; 4 Department of Orthopedic Surgery, the First Affiliated Hospital of Chongqing Medical University, Chongqing, 400016 China; 5 Center for Energy Metabolism and Reproduction, Shenzhen Institutes of Advanced Technology, Chinese Academy of Sciences, Shenzhen, 518055 China

**Keywords:** Metabolic syndrome, Pathogenesis

## Abstract

Although aging has traditionally been viewed as the most important risk factor for osteoarthritis (OA), an increasing amount of epidemiological evidence has highlighted the association between metabolic abnormalities and OA, particularly in younger individuals. Metabolic abnormalities, such as obesity and type II diabetes, are strongly linked to OA, and they affect both weight-bearing and non-weight-bearing joints, thus suggesting that the pathogenesis of OA is more complicated than the mechanical stress induced by overweight. This review aims to explore the recent advances in research on the relationship between metabolic abnormalities and OA risk, including the impact of abnormal glucose and lipid metabolism, the potential pathogenesis and targeted therapeutic strategies.

## Introduction

### Osteoarthritis

Osteoarthritis (OA) is a chronic and degenerative joint disease that is prevalent among elderly individuals. It is characterized by cartilage destruction and persistent pain, which can severely impact quality of life. The global incidence of OA has been increasing due to the aging population and other contributing factors, such as metabolic disorders, with an estimated conservative number of 250 million OA patients worldwide^1,2^. The pathological changes of OA are multifaceted, including synovitis, cartilage degeneration, subchondral bone thickening, osteophyte formation, ligament degeneration, meniscus injury, and structural changes in the surrounding muscles^2–5^. Unfortunately, current treatment options for OA can only relieve pain and manage symptoms, rather than stopping or reversing disease progression. For most severe cases, joint replacement surgery may be needed, which is expensive, invasive and carries significant risks^6,7^. Thus, finding new strategies to prevent OA development or improve OA treatment is of utmost importance. Further research is needed to better understand the underlying mechanisms of the disease and to develop new therapies that can effectively treat OA.

The etiology of OA remains elusive, and recent studies have demonstrated that OA is a multifaceted disease influenced by various pathogenic factors^8–10^. Aging and overweight are well-known risk factors for OA, but metabolic homeostasis imbalance has also been implicated in its pathogenesis^11^. Inflammation plays a crucial role in the progression of OA, which often originates from the adipose tissue in the joint cavity^12^. In a cohort study evaluating the incidence of obesity and OA among 1 764 061 subjects, researchers found that the risk of knee osteoarthritis (KOA) in obese individuals was more than three times higher than the risk in healthy individuals^13^. In patients with obesity, adipose tissue produces adipokines such as leptin, lipocalin, resistin and endolipoproteins, as well as inflammatory cytokines including tumor necrosis factor-alpha (TNF-α), interleukin 1 (IL-1) and interleukin 6 (IL-6)^14–16^. These mediators are released from local or systemic adipose tissue as the result of joint trauma or overuse and can significantly impact the development and progression of OA^17^. Further research is needed to fully understand the complex mechanisms underlying the relationship between obesity, inflammation and OA, which may lead to the development of novel therapeutic strategies.

### Metabolic abnormalities

Metabolic abnormalities encompass a range of conditions, such as obesity, hypertension, dyslipidemia, hyperglycemia, and insulin resistance. These risk factors not only contribute to the onset and progression of OA but also potentially increase the likelihood of developing cardiovascular disease. A multicenter study revealed that individuals with chronic obesity are prone to damage in the medial knee cartilage, leading to the development of OA^18^. Moreover, there exists a direct association between weight gain and the risk of KOA, as evidenced by a 35% increase in the risk for every five-unit increase in body mass index (BMI)^19^. It is thus clear that obesity plays a significant role in the manifestation of OA. Nevertheless, subsequent studies have demonstrated that hand osteoarthritis (HOA) can also occur in non-weight-bearing areas of obese individuals, suggesting that OA is not solely influenced by mechanical load but is also associated with metabolic abnormalities^20^. Chronic hyperglycemia and insulin resistance create an environment that promotes the development of OA, as elevated glucose levels induce the synthesis of proinflammatory cytokines and matrix metalloproteinases in joint tissues, leading to damage to human chondrocytes and the subsequent development of OA^21,22^. Additionally, dyslipidemia and hypertension have also been identified as causative factors for OA^16,23^. Consequently, metabolic disorder is one of the key risk factors for OA development and progression.

### Association between metabolic disorders and OA risk

A cross-sectional study aimed to examine the association between dietary glycemic index (GI), dietary glycemic load (GL), and knee OA among Korean adults, and the results showed a significant positive association between dietary GI and symptomatic KOA in women^24^. In addition, a study investigated the relationship between the Mediterranean diet (with lower GI) and prevalence of OA of the knee in a large cohort from North America, where the researchers found that individuals who had higher adherence to the Mediterranean diet usually had a lower risk of KOA^25^.

Metabolic abnormalities not only augment the susceptibility of OA but also impede the functional recovery of joint replacement surgery. A clinical follow-up study conducted in Canada found that metabolic abnormalities adversely impact patient functionality subsequent to joint replacement surgery, especially in the case of hip surgery^26^. Furthermore, in another cross-sectional investigation examining the relationship between metabolic syndrome and symptomatic KOA, the results demonstrated a positive correlation between the severity of symptomatic KOA and the metabolic syndrome accumulation factor^27^.

## The adverse effects of diabetes and obesity on OA

OA and diabetes often coexist due to their high prevalence and common risk factors. Nearly 47.3% of individuals with diabetes had some form of arthritis^28^. The negative impact of diabetes on joints could be explained by the induction of oxidative stress, pro-inflammatory cytokines, chronic high glucose concentration and insulin resistance.

### Chronic high glucose concentration

In individuals with type 2 diabetes, elevated levels of blood glucose result in the generation and accumulation of advanced glycation end products (AGEs) due to the maintenance of prolonged hyperglycemia^29^. This process promotes matrix stiffness. Collagen, which is a key component of various connective tissues, exhibits an exceptionally low turnover rate, rendering it susceptible to modifications by AGEs. Additionally, AGEs bind to the receptor for AGEs (RAGE) on the chondrocyte membrane, initiating intracellular signaling that leads to the overexpression of proinflammatory and prodegradative mediators^23,30^. In human OA chondrocytes, the specific binding of AGEs to RAGE activates the MAPK signaling pathway, thereby enhancing the expression of IL-6 and IL-8^31^. This, in turn, exacerbates the inflammatory response within chondrocytes^31^. Activation of RAGE by AGEs in articular chondrocytes prompts an increase in matrix catabolism in articular cartilage, ultimately contributing to the development of OA^32^. Moreover, elevated levels of AGEs in human articular chondrocytes further impede the turnover of the extracellular matrix in articular cartilage, promote cartilage degradation and diminish proteoglycan synthesis^33^. Studies have reported significantly higher levels of AGEs in the cartilage of OA patients than in healthy individuals^32^. Inhibition of the JAK/STAT3 signaling pathway following RAGE activation by AGEs led to a decrease in the expression of matrix metalloproteinase 13 (MMP13) and a disintegrin and metalloproteinase with thrombospondin motifs 5 (ADAMTS5), resulting in an increase in the synthesis of type II collagen (Col-2) in chondrocytes^34^. Researchers have also observed that incubation of rabbit chondrocytes with AGEs upregulates reactive oxygen species (ROS) expression, impairs mitochondrial function and induces chondrocyte death^35^. The accumulation of AGEs renders the collagen network in articular cartilage fragile, thereby increasing the risk of developing OA^36^.

### Proinflammatory cytokines

Proinflammatory cytokines, including IL-1β, TNF-α, and IL-6, are primarily synthesized by activated macrophages and play a crucial role in the inflammatory response associated with OA^37,38^. Epidemiological studies have identified diabetes and obesity as contributing factors to the development of OA^23,39^. These conditions induce a local or systemic state of low-grade inflammation in the human body. Hyperglycemic environments and adipose tissue have been shown to increase the in vivo expression of proinflammatory factors, such as IL-1β, IL-6 and TNF-α. This upregulation further activates the nuclear factor-κB (NF-κB) signaling pathway, increases the catabolic activity of articular chondrocytes and promotes degradation of the extracellular matrix (ECM), ultimately leading to OA progression^4,16,17,40–42^. In a stress-induced mouse model of OA, researchers observed that the group fed a high-fat diet exhibited significantly higher serum levels of TNF-α and more severe cartilage damage than the control group. However, the Toll-like receptor-5-deficient (*Tlr5* KO) mouse group, also on a high-fat diet, displayed significantly lower serum levels of IL-6 than the other groups, suggesting that obesity increases the expression of proinflammatory factors, thereby aggravating OA progression^43^. Another study demonstrated a significant elevation of IL-1β in the serum of mice fed a high-fat diet, with IL-1β inducing an inflammatory response in OA chondrocytes through activation of the NF-κB signaling pathway^44,45^. Notably, some studies have reported that metformin, a medication used to treat diabetes, not only reduces body mass index (BMI) in obese individuals but also decreases the rate of joint replacement surgery in patients with OA^46–48^. In murine studies conducted by Li et al., TNF-α and IL-1β markedly increased the mRNA expression levels of matrix metalloproteinase 3 (MMP3), MMP13, metalloproteinase with thrombospondin motifs 4 (ADAMTS4) and ADAMTS5 in primary articular chondrocytes^49^. However, the addition of metformin effectively suppressed the expression of MMP13 and MMP3 induced by TNF-α and IL-1β, and it was further revealed that metformin exerted its inhibitory effects on MMP13 and MMP3 expression by attenuating the catabolic responses induced by inflammatory cytokines and promoting the expression of anabolic genes, thereby safeguarding articular chondrocytes^49^. Consequently, inhibiting the expression of proinflammatory factors through weight reduction or controlling diabetic blood glucose levels can confer positive therapeutic outcomes for the treatment of OA^22,50^.

### Reactive oxygen species

Reactive oxygen species (ROS) are highly reactive molecules that contain oxygen and can cause damage to cells. Oxidative stress and mitochondrial dysfunction are known to be the primary sources of ROS. Extensive research has shown that obesity and diabetes can induce elevated levels of ROS in the body^51–54^. Adipose tissue and high blood glucose levels create a proinflammatory environment, leading to an increase in M1-type macrophages and proinflammatory cytokines such as IL-1, TNF-α, and IL-6. These factors contribute to tissue damage and further stimulate the secretion of proinflammatory cytokines, exacerbating oxidative stress and mitochondrial dysfunction; consequently, tissues experience heightened levels of ROS^55,56^. Overproduction of ROS is a contributing factor to the development and progression of OA^57^. In OA chondrocytes, excessive ROS production activates the MAPK and NF-κB signaling pathways, disrupting the balance between cartilage catabolism and anabolism. This imbalance leads to increased catabolism of articular cartilage, synovial inflammation and subchondral bone thickening^56,58^. Studies have demonstrated that incubation of chondrocytes with H_2_O_2_ results in increased ROS production, chondrocyte death^59^, degradation of chondrocyte ECM and inhibition of proteoglycan synthesis, which accelerate OA progression^60,61^. However, the use of ROS inhibitors or scavengers can slow cartilage loss. Jin et al. conducted experiments in a surgically induced mouse model of knee OA and found that intraperitoneal injection of ROS inhibitors significantly reduced the severity of cartilage damage in the knee joints^62^. Additionally, ROS inhibitors decreased the mRNA levels of MMP13 and ADAMTS5 in OA chondrocytes while increasing the mRNA levels of Col-2 and Aggrecan^62^. Thus, ROS inhibitors reduce cartilage damage by inhibiting ROS transduction in the MAPK and NF-κB signaling pathways. In the IL-1β-induced human synovial explant of the OA model, the level of the oxidative stress marker 8-OHdG exhibited a fourfold increase, and there was also a significant elevation in MMP13 and ADAMTS5 expression^63^. Notably, the addition of antioxidants resulted in a significant decrease in the expression of MMP13 and ADAMTS5, indicating that ROS inhibitors may possess the potential to alleviate synovial inflammation in OA^63^. Lu et al. employed an ACLT surgery-induced rat model of KOA and discovered that the ROS scavenger known as black phosphorus nanosheets (BPNSs) effectively eliminated intracellular ROS while concurrently maintaining cartilage morphology and impeding the reduction of subchondral bone volume in KOA^64^. Furthermore, it was observed that increased ROS levels in OA chondrocytes could hinder the mitochondrial respiratory chain and give rise to mtDNA mutations. This ROS-induced mtDNA damage subsequently prompted enhanced expression of MMP1 and MMP3 in chondrocytes, thereby further increasing the progression of OA^58,65^. Consequently, reducing ROS production exerts a significant effect on the retardation of articular chondrocyte senescence or the mitigation of associated damage^66^.

### Insulin resistance of the diabetic synovial membrane

Insulin resistance refers to the diminished physiological response of specific organs or tissues in the body to normal insulin levels, necessitating higher insulin concentrations to maintain normal insulin function^67^. The presence of insulin resistance underlies the development of type 2 diabetes, and its impact on KOA severity is significantly more pronounced in individuals with type 2 diabetes than in nondiabetic KOA patients^68^; notably, diabetes induces more severe synovial inflammation or synovial thickening in both diabetic mice and patients with KOA^69–71^. Extensive expression of insulin receptors (IRs) has been observed in the synovium of both mice and humans^69^. Moreover, obese KOA patients with type 2 diabetes exhibit elevated levels of TNF in their synovium, whereas this elevation is not observed in obese KOA patients without diabetes^69^. Fibroblast-like synoviocytes (FLS) respond to increased TNF by upregulating the production of IL-1, TNF-α, IL-6, bone morphogenetic protein 2 (BMP-2), ADAMTS4 and MMP13^69,72^. Hamada et al. isolated synoviocyte fibroblasts from KOA patients without diabetes and found that insulin inhibited the induction of TNF-mediated cytokines, growth factors and proteases^69^. However, in diabetic KOA patients, the inhibitory effects of insulin on TNF-induced cytokines, growth factors and protease production are diminished due to impaired signaling by insulin-resistant synovial IRs^69^. BMP-2, in conjunction with cytokines and proteases, further intensifies the progression of OA, as it promotes the development of osteochondritis dissecans^73^. Additionally, chronic hyperglycemia triggers oxidative stress, proinflammatory cytokines and excessive production of AGEs within joint tissues. These factors induce the production of vascular endothelial growth factor (VEGF) and aggravate the synovial inflammatory response in human synoviocytes through activation of the RAGE-NF-κB pathway, ultimately leading to joint damage in OA patients^30,74,75^. In diabetic patients, insulin resistance promotes the progression of OA by impairing the protective and anti-inflammatory effects of insulin within the synovium. Studies have also identified another hepatic metabolic factor, LECT2, which is highly expressed in the liver^76^. LECT2 mediates glucose metabolism and obesity-related insulin resistance^76^. In a healthy male population, LECT2 concentrations in the blood increased with the intake of a high-fat diet, suggesting that LECT2 has an important effect on metabolic homeostasis in the body^77^. The researchers found that LECT2 appeared at high expression levels in OA patients and elderly individuals, suggesting that LECT2 may be involved in OA^78^. Further studies are needed in the future to determine the pathological mechanisms of LECT2 in OA.

**Fig. 1:**
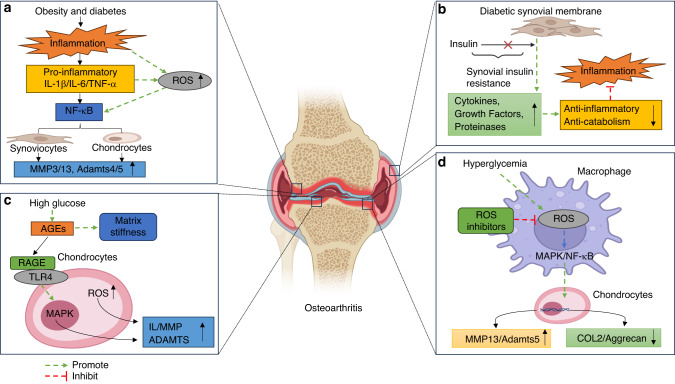
Effect of Glucose Metabolism on Osteoarthritis. **a** Obesity and diabetes lead to increased pro-inflammatory cytokines IL-1β, IL-6, TNF-α, and ROS, activate the NF-κB pathway and promote the expression of MMP3, MMP13, ADAMTS4 and ADAMTS5. **b** Normal synovial cells can receive insulin, which acts as an anti-inflammatory. Insulin-resistant synovial cells are insensitive to insulin, resulting in increased levels of the proinflammatory cytokines IL-1β, IL-6, growth factor BMP-2, protease MMP13 and ADAMTS4, aggravating the degree of osteoarthritis. **c** Hyperglycemia induces the production of AGEs, which promote cartilage matrix stiffness as well as ROS production. AGEs bind to RAGE, activate the MAPK signaling pathway in chondrocytes, and promote the production of IL, MMP and ADAMTS. **d** Hyperglycemia promotes the expression of ROS and M1 macrophage activation. ROS activate the MAPK and NF-κB pathways, promote MMP13 and ADAMTS5 and inhibit the expression of Col-2 and Aggrecan. ROS inhibitors inhibit the activation of this pathway. IL-1β interleukin-1β, IL-6 interleukin-6, TNF-α tumor necrosis factor-α, ROS reactive oxygen species, NF-κB nuclear factor-κ-gene binding, MMP3 matrix metalloproteinases 3, MMP13 matrix metalloproteinases 13, ADAMTS4 a disintegrin and metalloproteinase protein 4, ADAMTS5 a disintegrin and metalloproteinase protein 5, BMP-2 bone morphogenetic protein-2, AGEs advanced glycation end products, RAGE receptor for advanced glycation end products, MAPK mitogen-activated protein kinase, Col-2 Collagen-2

In conclusion, obesity- and diabetes-induced hyperglycemia, oxidative stress, inflammatory response and insulin resistance suggest that disturbance in glucose metabolism may contribute to metabolic OA (Fig. 1).

## Lipid metabolism and OA

The process of lipid metabolism includes the synthesis and degradation of lipids in the cell, which is critical for the proper functioning of living organisms. Lipid metabolism involves the digestion, absorption, synthesis, storage and breakdown of fats and the transport of various synthesized substances throughout the body to meet physiological needs, such as the construction of cell membranes. The action of various enzymes and bile salts hydrolyzes fats into glycerol, fatty acids and other substances. Lipids are absorbed through two mechanisms: triglycerides composed of medium-chain and short-chain fatty acids are emulsified and directly absorbed into the blood, while triglycerides containing long-chain fatty acids combine with apolipoproteins and cholesterol to form chylomicrons, which are ultimately absorbed into the blood via the lymphatic system^79^. After metabolism and absorption, fats are divided into four lipid groups: triglycerides, phospholipids, cholesterol, and plasma lipoproteins. Other substances in the body control the four lipid groups and change them into substances needed for various biochemical processes in the organism. However, when the homeostatic balance of lipid metabolism is disturbed, it will predispose the body to diseases, including OA^11^. Numerous studies have found that patients with lipid metabolism disorders suffer from a higher risk of OA^16,80,81^. In a national study in the United States, which focused on the prevalence of OA and metabolic syndrome in subjects with OA and the general population without OA, the results showed that the prevalence of OA was more than twice as high in individuals with metabolic disorders as in the control population^82^. This review examines the relevance of important lipid metabolites in the development of OA and explores the underlying mechanisms of lipid metabolism disorders in OA pathology, thus providing new insights into the treatment of metabolic OA.

### Effects of triglyceride metabolism on OA

A triglyceride is an ester consisting of glycerol and three fatty acids. Elevated levels of serum triglycerides are also a risk factor for the progression of OA^83^. During moderate- to low-intensity exercise, the breakdown of triglycerides can provide most of the energy needed by the exercising muscles. However, when there is excessive fat intake over a prolonged period, the breakdown and metabolism of fats can exceed the body’s capacity, resulting in the accumulation of fatty acids. Triglycerides stored in adipose tissue are gradually hydrolyzed into glycerol and free fatty acids (FFAs) by lipases and released into the bloodstream. Fatty acids can be classified as saturated fatty acids (SFAs), monounsaturated fatty acids (MUFAs) and polyunsaturated fatty acids (PUFAs) based on the length of their carbon chain and the number of double bonds.

Excessive lipid intake leads to an increased breakdown and metabolism of triglycerides, resulting in elevated levels of SFAs in the blood^84^. In vitro, studies have shown that treating cartilage explants with SFAs increases the expression of glycosaminoglycans (GAGs), IL-6 and poly (ADP-ribose) polymerase (PARP) and decreases the viability of chondrocytes in the top layer of the explants^85^. SFAs also induce the upregulation of the autophagy markers microtubule-associated protein and the expression of the p65 protein and activate autophagy and NF-κB signaling pathways in C28/I2 chondrocytes^86^. In an SFA-induced OA chondrocyte model, IL-1β and MMP13 mRNA expression was increased, while Col-2 and Sox9 mRNA expression was decreased and chondrocyte glucose uptake was reduced^87^. After feeding Wistar rats an SFA-containing diet for 16 weeks, the results of immunohistochemical (IHC) staining revealed an increase in MMP13 and Col-X expression and a decrease in aggrecan (ACAN) expression in the joint cartilage, and the results of micro-CT showed a decrease in the bone volume fraction of the tibia^84^. A clinical study of the relationship between diet and OA progression in 2 092 patients with OA showed that as dietary SFA levels in OA patients increased, the width of the joint space decreased by 0.26 mm, 0.27 mm, 0.31 mm and 0.35 mm at 12, 24, 36, and 48 months after feeding with a high SFA diet, respectively, suggesting that high levels of SFA intake may aggravate structural damage in KOA^88^.

There is relatively limited research on the relationship between MUFAs and the progression of OA. Gas chromatography‒mass spectrometry (GC‒MS) has been used to determine the fatty acid composition of the infrapatellar fat pad in a rabbit model of OA caused by anterior cruciate ligament transection (ACLT). This procedure led to a decrease in the amount of MUFAs in the knee joint, but the link between MUFAs and OA is still not clear^89^. However, the finding of an in vitro study demonstrated that administration of MUFAs may be able to inhibit cartilage degradation^90^. This may explain why the proportion of MUFAs is decreased in patients with knee joint OA. In a TNF-α-induced chondrocyte injury model, MUFAs were found to inhibit the mRNA expression of prostaglandin-endoperoxide synthase-2 (PTGS2) and matrix metalloproteinase 1 (MMP1), thus inhibiting cartilage degradation^90^. In a clinical study, the synovial fluid of 23 OA patients undergoing total knee replacement surgery was analyzed, and it was found that the level of MUFAs in the synovial fluid of the OA group was higher than that in the non-OA group. However, the mechanism underlying the relationship between MUFAs and OA cartilage is still unclear^91^. The different results obtained from observational studies may be due to differences in the selected study populations. Further research is needed to clarify the relationship between MUFAs and OA.

**Fig. 2:**
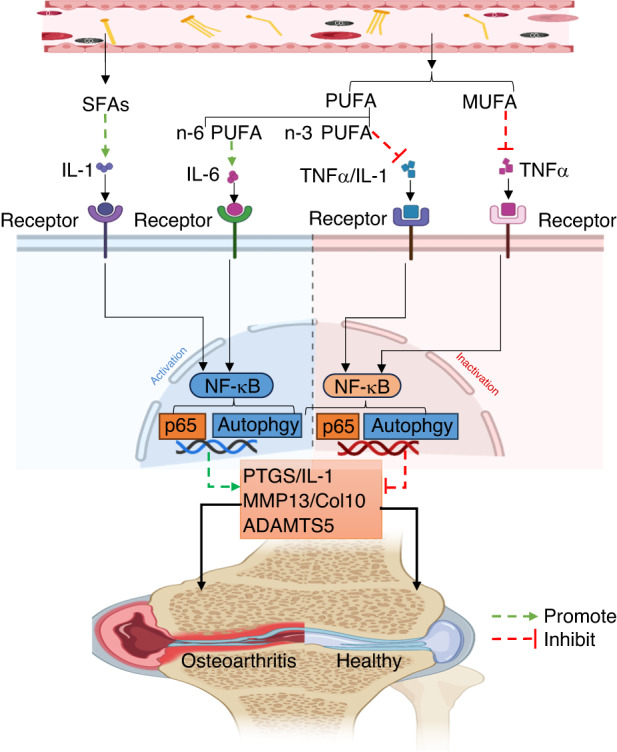
The mechanism of fatty acids in osteoarthritis. Excess SFAs and n-6 PUFAs in the blood directly or indirectly activate the NF-κB pathway by binding to the corresponding receptors, promoting the expression of PTGS, IL-1, MMP13, ADAMTS5, and COL10 and aggravating OA. MUFAs and n-3 PUFAs can inhibit the expression of proinflammatory factors such as TNF-α and IL-1 and block the activation of the MAPK and NF-κB pathways. SFAs saturated fatty acids, n-6 PUFAs omega-6 polyunsaturated fatty acids, NF-κB nuclear factor-k-gene binding, PTGS prostaglandin-endoperoxide synthase, IL-1 interleukin-1, MMP13 matrix metalloproteinases 13, ADAMTS5 a disintegrin and metalloproteinase protein 5, Col-X Collagen-10, MUFA monounsaturated fatty acids, n-3 PUFA omega-3 polyunsaturated fatty acids, TNF-α tumor necrosis factor-α, MAPK mitogen-activated protein kinase

Polyunsaturated fatty acids (PUFAs) are a unique class of bioactive compounds that play important physiological roles in the human body. PUFAs can be classified into omega-3 (n-3) and omega-6 (n-6) PUFAs based on the position of their double bonds. N-3 PUFAs include eicosapentaenoic acid (EPA) and docosahexaenoic acid (DHA), while n-6 PUFAs include linoleic acid (LA) and arachidonic acid (AA)^92,93^. N-3 and n-6 PUFAs are precursors for the synthesis of eicosanoids, and the balance of these two molecules in the body plays an important role in stabilizing cell membrane function, regulating gene expression and maintaining cytokine function. In healthy adult chondrocytes cultured with n-6 PUFAs, the secretion of IL-6 was significantly increased^94^. AA increases PEG production and ADAMTS mRNA expression in canine chondrocytes^95^. In a male mouse fed a high n-6 PUFA diet, n-6 PUFAs cause the progression of OA, prolong the wound healing time and increase the expression of inflammatory adipokines^96^. In a prospective cohort study of 5 328 participants, including 42% men, the plasma n-6 PUFA levels in male OA patients were positively correlated with joint effusion and knee structural damage levels^97^. SFAs and n-6 PUFAs can exacerbate cartilage structure damage by enhancing cell apoptosis and the expression of cartilage degradation-related genes^85,97^. Studies have shown that increasing n-3 PUFAs and decreasing n-6 PUFAs in the bodies of transgenic fat-1 mice can significantly alleviate cartilage destruction and osteophyte formation in the mouse OA model, reduce the expression of MMP13 and ADAMTS5 in joint cartilage, and stop the loss of chondrocytes and extracellular matrix^98^. Another study found that n-3 PUFAs significantly reduced the mRNA expression of ADAMTS4, ADAMTS5, MMP3, MMP13, COX-2, IL-1β and TNF-α when bovine chondrocytes were incubated with n-3 PUFAs, while n-6 PUFAs had no effect on the mRNA expression of cartilage degradation-related genes and inflammatory cytokines^99^. The researchers collected plasma from 167 patients with knee joint OA and found that OA patients with a high n-6:n-3 PUFA ratio had lower pain thresholds and more obvious limitations in joint movement and activities^100^. In another clinical trial, symptomatic knee OA patients who took different doses of n-3 PUFAs (fish oil supplements) experienced a reduction in clinical pain symptoms and an increase in joint functions and physical activities in the first year of treatment^101^. The WOMAC score showed that OA patients treated with a low dose of n-3 PUFAs had greater improvement in pain levels and physical functions after 2 years of treatment^101^ (Fig. 2). We have summarized the fatty acid species and their effects on OA in Table 1.

**Table 1:** Fatty acids and the development of OA

Type of fatty acid	Type of study	Role in the pathogenesis of OA	Potential relationship with OA	Reference
SFA	In vitro model	Promotes IL-6 release, and ECM degradation, induces chondrocytes death.	Promote OA	^85^
	In vitro model	Increased activation of autophagy and NF-κB signaling pathways.		^86^
	In vitro model	Increased IL-1β and MMP13 expression, decreased the expression of collagen-II and Sox9.		^87^
	Rat model	Increased MMP13 and Col-X expression, decreased bone volume fraction and ACAN expression.		^84^
	Clinical trial	Reduce the width of the knee gap and aggravate the damage to the cartilage structure of the joint.		^88^
MUFA	In vitro model	Inhibit expression of PTGS2 and MMP1.	Inhibit OA	^90^
	Rabbit model	Inhibit cartilage destruction.		^90^
N-6 PUFA	In vitro model	1. Increased expression of IL-6. 2. Upregulation of PEG and ADAMTS expression.	Promote OA	^94,95^
	Mouse model	Prolongs the time to wound healing, and increases the expression of inflammatory adipokines.		^96^
	Clinical trial	Exacerbates damage to the structure of the knee joint.		^97^
N-3 PUFA	In vitro model	Reduces the expression of ADAMTS4, ADAMTS5, MMP3, MMP13, COX-2, IL-1β, and TNF-α.	Inhibit OA	^99^
	Mouse model	Reduces the expression of MMP13 and ADAMTS5, alleviate cartilage destruction and osteophyte hyperplasia.		^98^
	Clinical trial	Reduces clinical pain symptoms and improves motor function.		^101^

### Effects of phospholipid metabolism on OA

Phospholipids are the main components of biological membranes and can be divided into two classes: glycerophospholipids and sphingolipids (SM). Glycerophospholipids are the most abundant type of phospholipids in the body and can participate in cell membrane recognition and signal transduction. Based on LC‒MS plasma lipidomics analysis, Pousinis et al. found 24 lipid spectra differences between the plasma of DMM-induced OA mice and sham-operated mice. The significantly higher SM in the plasma was positively correlated with the degree of DMM-induced joint cartilage injury^102^. Phosphatidylinositol-4-phosphate 5-kinase type γ (PIP5K1c) is a lipid kinase that catalyzes the synthesis of phosphatidylinositol 4,5-bisphosphate (PIP2) and participates in various cellular processes. It has been reported that mice with *Pip5k1c* gene deletion exhibit a variety of spontaneous OA pathological phenotypes, including cartilage degeneration, surface fissures, subchondral sclerosis, meniscus deformation, synovial hyperplasia and osteophyte formation^103^. These findings suggest that *Pip5k1c* expression in chondrocytes plays a critical role in maintaining joint tissue homeostasis.

In the pathological process of OA, the lubrication of synovial joints could be influenced by mechanical and molecular factors and by changes in synovial fluid. Synovial fluid can reduce joint wear and maintain tissue homeostasis. In healthy individuals, an effective lubricating layer is formed among the surfaces of cartilage and other joint tissues, and changes in the structure and composition of this layer could lead to lubrication abnormalities and dysfunction of joint tissues and OA symptom progression^104^. Researchers collected synovial samples from 13 OA patients who underwent knee replacement surgery for lipid measurements and found that the spatial distribution of glycerophospholipids was correlated with hypertrophic, inflamed or vascularized synovial regions^105^. Kosinska et al. used lipidomics and electrospray ionization tandem mass spectrometry methods to analyze synovial fluid (SF) samples from 17 early OA patients, 13 late OA patients, 18 RA patients, and 9 control donors postmortem and identified the following phospholipid categories in SF: phosphatidylcholine (PC), lysophosphatidylcholine (lysoPC), phosphatidylethanolamine, phosphatidylethanolamine-derived aldehyde phospholipid, phosphatidylglycerol, phosphatidylserine, sphingolipids and ceramides. Compared to the median PC concentration in the SF of the control group, the median PC concentration in the SF of early OA is 2.7-fold higher than that of the control, and the median PC concentration in the SF of late OA is 5.4-fold higher than that of the control^106^. In a clinical cohort study, investigators collected serum from 24 patients with KOA for metabolomics analysis and evaluated the volume of cartilage loss between baseline and 24 months using magnetic resonance imaging (MRI). The results showed that the increased serum ratio of lysoPC to PC was associated with the volume of lateral compartmental cartilage loss in the knee joint and the increase in the joint degradation markers COMP and MMP1^107^. A recent study has demonstrated the differences in SF phospholipidomics between knee OA patients and non-OA patients, with higher levels of PC, phosphatidylserine and phosphatidylinositol in the SF of OA patients than in non-OA control subjects^108^. The plasma ratio of lysoPC to PC also significantly increased in KOA patients^109^. This ratio could be used to predict OA risk, disease progression and treatment response^110^. This finding suggests that increased conversion of PC to lysoPC, which is catalyzed by phospholipase A2 (PLA2), is associated with OA progression^111^. Pruzanski et al. found that the concentration of PLA2 in cartilage is higher than that in synovium, suggesting that cartilage may be the main source of PLA2 production^112^. PLA2 has also been found to play a central role in OA inflammation^113^.

### The impact of cholesterol metabolism on OA

Cholesterol in the human body mainly comes from two sources: endogenous biosynthesis and intestinal absorption. The liver is the major organ for cholesterol synthesis, and other tissues in the body can also synthesize cholesterol^114^. It has been reported that cholesterol can alter Indian hedgehog (IHH) activity to regulate the development of articular cartilage, suggesting that cholesterol plays an important role in cartilage development^115^. Numerous studies have shown that promoting cholesterol efflux or increasing cholesterol metabolism may help protect chondrocytes from the influence of inflammation^116–118^. Nuclear receptors are one of the most abundant transcriptional regulators in animals, as they play important roles in metabolism, differentiation, reproductive development and homeostasis maintenance. Reports have shown that nuclear receptors, such as liver X receptor (LXR), peroxisome proliferator-activated receptor (PPAR) and retinoic acid-related orphan receptor α (RORα), play a key role in the transcriptional regulation of lipid metabolism^119–121^. They are closely related to the occurrence and development of metabolic OA^122^.

LXR regulates cholesterol efflux-related genes and plays a key role in the transcriptional regulation of lipid metabolism-related genes as a member of the nuclear hormone receptor superfamily. By activating reverse cholesterol transport (RCT), LXR promotes the conversion of cholesterol into bile acids in the liver, thus protecting the body from hypercholesterolemia^79,123,124^. Reports have shown that the expression of cholesterol efflux genes in OA patients is significantly reduced and that the expression level of LXR is positively correlated with the expression of cholesterol efflux genes^116,125^. In a chondrocyte OA model, treatment with the LXR agonist TO-901317 significantly increased the mRNA and protein levels of cholesterol efflux genes ApoA1 and ABCA1 and reduced lipid deposition in OA chondrocytes, suggesting that changes in LXR levels in chondrocytes may be a contributing factor in the regulation of dynamic OA development^116^. It has also been found that LXR activation regulates the expression of lipid homeostasis-related genes in chondrocytes and the free cholesterol content in chondrocytes through the LXR–Srebp1–Scd1 axis^120^. After treatment with IL-1β and TNF-α, articular chondrocytes from OA patients showed significantly reduced LXR expression, leading to polysaccharide protein degradation through negative feedback regulation of the activated NF-κB signaling pathway, suggesting that decreased LXR expression levels could promote OA development^126^. In addition, LXR activation can significantly reduce the expression of proinflammatory cytokines such as TNF-α, COX-2, IL-1β, MMP9 and iNOS^127^ and inhibit Toll-like receptor-mediated inflammatory responses by promoting cholesterol efflux in macrophages^128^. Vaspin can inhibit miR-155 expression in rat chondrocytes and promote cholesterol efflux. When Vaspin expression is reduced, LXRα and other cholesterol efflux-related genes are inhibited in chondrocytes, leading to cholesterol accumulation in chondrocytes and worsening OA progression^129^. Reports have shown that metformin can activate the AMPK/SIRT1 signaling pathway to reverse IL-1β-induced extracellular matrix degradation in chondrocytes^130,131^. Activation of the AMPK/SIRT1 pathway upregulates LXRα expression, thereby promoting cholesterol efflux in chondrocytes^130,131^. AMPK signaling could interact with many signaling pathways that may be involved in OA occurrence and progression. For example, it has been shown that metformin inhibits β-catenin^S552^ phosphorylation and nuclear translocation^132^. The AMPK activators metformin and berberine inhibit OA progression^49,133,134^. In an IL-1-treated chondrocyte model, researchers observed that IL-1β significantly downregulated the mRNA and protein expression of cholesterol efflux-related factors ABCA1, ApoA1 and LXR in chondrocytes. Resveratrol (RES) can activate the SIRT1/FoxO1 signaling pathway to promote LXRα expression, reduce cholesterol accumulation in chondrocytes and delay OA progression^135^.

**Fig. 3:**
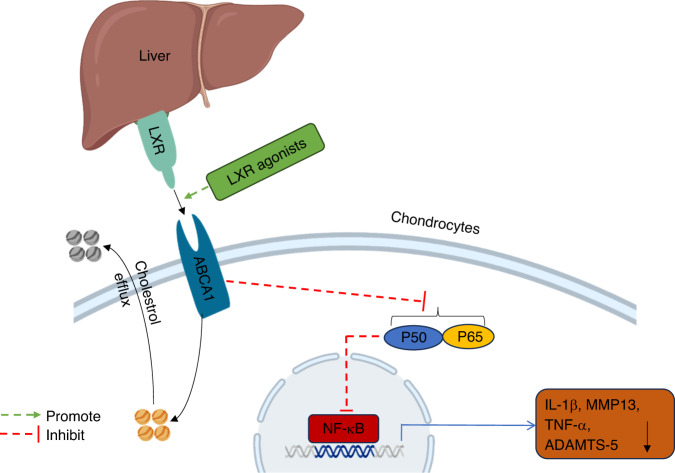
The role of liver nuclear receptor LXR in osteoarthritis. The liver nuclear receptor LXR binds to ABCA1, promotes the efflux of cholesterol in chondrocytes, inhibits the activation of the NF-κB pathway and reduces the expression of IL-1β, TNF-α, MMP13 and ADAMTS5. LXR agonists can promote LXR expression and strengthen the above two pathways. LXR liver X receptor, ABCA1 ATP-binding cassette transporter A1, NF-κB nuclear factor-k-gene binding, IL-1β interleukin-1β, TNF-α tumor necrosis factor-α, MMP13 matrix metalloproteinases 13, ADAMTS5 a disintegrin and metalloproteinase protein 5

Due to the inability of peripheral cells to degrade cholesterol, excessive cholesterol efflux is the only way to eliminate cholesterol from these cells. When the LXR transcription factor is activated, it binds to the promoter sequence of the ABCA1 gene. ABCA1 acts as a lipid pump to efflux cholesterol and phospholipids from osteoarthritic chondrocytes to ApoA1 (Fig. 3), generating new high-density lipoprotein (nHDL) particles^116,136,137^. Lecithin-cholesterol acyltransferase (LCAT) catalyzes the conversion of free cholesterol to cholesterol esters to form mature HDL, which enters the liver through bile secretion and fecal excretion, reducing cholesterol levels in chondrocytes^138^. Currently, cholesterol efflux agonists have an important impact on inhibiting metabolic OA progression. LCAT deficiency directly affects the normal physiological function of HDL. Researchers found that *LCAT*^*-/-*^ mice fed a high-fat diet for 24 weeks developed OA pathology, indicating that the abnormal physiological function of HDL led to the occurrence of an OA phenotype in mice^139^. There are also reports showing that high levels of HDL have a certain preventive effect on OA progression^140^. LCAT is mainly produced and secreted by the liver, and upregulation of LCAT expression has been shown to enhance the reverse cholesterol transport (RCT) process in mice with hepatic osteodystrophy, thereby alleviating bone loss^141^.

PPAR plays an important role in lipid metabolism, the inflammatory response, and cell apoptosis^142,143^. PPAR regulates many metabolic processes in cells, including the three subtypes PPARα, PPARγ and PPARδ. Increasing evidence suggests that PPAR is involved in the occurrence and development of OA and is closely related to the regulation of lipid metabolism disorders and OA.

PPARα is present in chondrocytes, endothelial cells and hepatocytes and exhibits anti-inflammatory effects. In aging and surgically induced OA mouse models, it has been shown that the numbers of PPARα-positive chondrocytes decreased gradually in the cartilage with aging and OA progression^144^. Further IHC analysis showed that PPARα expression in the cartilage of patients with KOA was significantly lower than that in the non-OA individuals, suggesting that PPARα plays an important role in the homeostatic regulation of chondrocytes^144^. Researchers have also divided the cartilage of OA patients into relatively healthy (non-OA) and severely damaged (OA) groups and found that the lipid deposition area in the OA group was significantly increased compared to that in the non-OA group. Additionally, the expression of PPARα in chondrocytes and cartilage of OA patients was significantly reduced. However, in OA chondrocytes treated with PPARα agonists, lipid deposition was significantly reduced, suggesting that PPARα may be involved in the development of OA by regulating lipid metabolism. The study also found that PPARα regulates the balance of joint cartilage homeostasis through the PPARα–ACOT12 pathway^145^. More recent studies have demonstrated that PPARα protects against articular cartilage damage in a mouse OA model by inhibiting the inflammatory response^146^. The mechanism of lipid deposition in articular cartilage and the pathogenesis of OA are not fully understood and require further in-depth investigation in the future.

PPARγ is highly expressed in cartilage tissue, and PPARγ agonists reduce inflammation and prevent cartilage degradation in OA animal models^147,148^. A study showed that PPARγ expression was downregulated during the progression of OA in STR/Ort mice, which was aggravated under inflammatory conditions in joint cartilage, leading to knee joint cartilage damage and osteophyte formation^149^. It has been reported that promoting cholesterol efflux through PPARγ-mediated pathways can promote extracellular matrix synthesis in OA chondrocytes in rabbits^118^. Studies have found that SUMO-modified PPARγ can improve lipid metabolism disorders in chondrocytes. In summary, PPARγ activation has a potential therapeutic effect on OA, but the specific mechanism of PPARγ-regulated lipid metabolism in decelerating OA progression has yet to be fully elucidated^150^.

Unlike PPARα and PPARγ, which have positive effects on cartilage degradation in OA, current research suggests that PPARδ activation could exacerbate OA progression^151^. When a PPARδ agonist (GW501516) was used to treat mouse chondrocytes, it was found that PPARδ activation significantly increased the mRNA expression of MMP2, MMP3, ADAMTS2 and ADAMTS5 in chondrocytes compared to that of nontreated cells, and the degree of fatty acid oxidation in chondrocytes was significantly increased^152^. It has also been shown that deletion of the *PPARδ* gene in chondrocytes helps alleviate OA symptoms induced by DMM surgery in mice, indicating that *PPARδ* deficiency has an inhibitory effect on OA development^152^. The potential mechanism of PPARδ-exacerbated OA could be that PPARδ promotes fatty acid oxidation in chondrocytes, which can induce the production of ROS and accelerate OA progression.

During cholesterol overload, cholesterol signaling activation promotes chondrocyte hypertrophy by upregulating the expression of the nuclear receptor RORα. RORα is a downstream target of the cholesterol metabolism pathway, the cholesterol-25-hydroxylase (CH25H)-oxysterol-7α-hydroxylase (CYP7B1) axis. Overexpression of RORα upregulates cartilage degradation-related genes and downregulates the expression of anabolic metabolic factors^153,154^. Cui et al. induced effective overexpression of CH25H in the joint by intra-articular injection of adenovirus-CH25H (Ad-CH25H) in mice and found that RORα upregulated the downstream mediators of cholesterol metabolism, leading to severe cartilage damage, osteophyte formation and thickening of the subchondral bone plate in mice, indicating that the CH25H-CYP7B1-RORα axis is involved in cholesterol metabolism and plays a role in the pathological process of OA^153^. In an ACLT-induced OA mouse model, RORα siRNA delivered by adenovirus was administered into the knee joint two weeks after surgery, the expression of Aggrecan and Col2a1 in joint cartilage was increased, and cartilage damage was partially reversed by RORα siRNA. Researchers found that RORα may regulate the progression of OA through the IL-6/STAT3 signaling pathway^155^. miR-10a-3p is an upstream target of CH25H. miR-10a-3p can reduce the production of cartilage degradation enzymes in chondrocytes under inflammatory conditions through regulation of the CH25H-CYP7B1-RORα axis and protect cartilage degeneration in a rat OA model^156^. RORα plays an important role in regulating the cholesterol metabolism pathway and can be a potential target for the treatment of metabolic OA.

Low-density lipoprotein receptor-related protein 3 (LRP3) not only regulates the steady state of blood lipids and fibrinolysis but also participates in the regulation of cholesterol metabolism. It has been shown that LRP3 can positively regulate the metabolism of extracellular matrix in chondrocytes, and the downregulation of LRP3 can activate the Ras signaling pathway and upregulate syndecan-4 protein levels, aggravating mouse knee joint cartilage degeneration^157^.

High cholesterol levels may play a key role in the pathogenesis of OA. Hypercholesterolemia can lead to atherosclerosis, causing ischemia and hypoxia in the corresponding blood supply area, resulting in inadequate energy supply in the joint tissue. Therefore, when cholesterol accumulates in the joint, the blood supply to the subchondral bone is insufficient, enhancing the insufficient oxygen and nutrient supply to the subchondral bone and worsening the pathological process of OA^158^. We summarize the functional components of cholesterol in OA and its associated molecular pathways in Table 2.

**Table 2:** The role of nuclear receptors associated with cholesterol metabolism in OA

Nuclear receptor type	Mechanism of action of nuclear receptors	Signaling pathways in OA	Reference
LXR	Increased expression of cholesterol efflux genes ApoA1 and ABCA1 and reduced lipid deposition in chondrocytes.	NA	^116^
	Promotes the expression of lipid homeostasis genes and reduces free cholesterol in chondrocytes.	LXR-Srebp1-Scd1 signaling pathway activation	^120^
	Promotes cholesterol efflux and inhibits inflammatory response.	NA	^128^
	Inhibits proteoglycan degradation.	NF-κB signaling pathway inhibition	^126^
	Inhibits expression of LXRα leads to the accumulation of cholesterol in cartilage.	NA	^129^
	Promotes the efflux of cholesterol inside chondrocytes.	AMPK/SIRT1 signaling pathway activation	^130,131^
	Reduces the accumulation of cholesterol in chondrocytes.	SIRT1/FoxO1 signaling pathway activation	^135^
PPARα	Reduces lipid deposition.	PPARα − ACOT12 signaling pathway activation	^145^
PPARγ	Reduces micro environmental inflammation and catabolism in articular cartilage.	NA	^149^
	Promotes extracellular matrix synthesis.	NA	^118^
	Inhibits abnormal lipid metabolism in chondrocytes.	NA	^150^
PPARδ	Increased expression of MMP2, MMP3, ADAMTS2 and ADAMTS5 in chondrocytes. Loss of PPAR-δ protects OA cartilage damage.	NA	^152^
RORα	1. Upregulation of cartilage degradation-related genes and downregulation of anabolic factors. 2. Exacerbates cartilage damage and subchondral bone thickening.	CH25H-CYP7B1-RORα axis	^153,154^
	Inhibition of RORα can promote the elevation of aggregate glycans and Col2a1 in articular cartilage.	IL-6/STAT3 signaling pathway	^155^

### The impact of plasma lipoprotein metabolism on OA

The structure of plasma lipoproteins is mostly spherical, consisting of a core of triglycerides and cholesterol esters, covered by a complex of lipids, phospholipids and free cholesterol molecules on the surface, ensuring the normal transport of lipids in the plasma. Lipoproteins can be classified into chylomicrons (CM), very-low-density lipoproteins (VLDL), low-density lipoproteins (LDL) and high-density lipoproteins (HDL) based on their density. HDL is mainly produced in the liver and is responsible for removing excess cholesterol from cell membranes. The plasma phospholipid cholesterol acyltransferase transfers fatty acid residues from phospholipids to cholesterol to produce cholesterol lipids, and then HDL transports cholesterol lipids to the liver, where excess cholesterol is converted into bile acids, maintaining the homeostasis of normal lipid metabolism in the body. Studies have found that metabolic syndrome and low HDL are associated with decreased medial tibial plateau cartilage volume, while insulin resistance, high waist circumference and low HDL-C are associated with tibial cartilage defects. Interventions targeting these pathogenic factors may prevent or delay knee joint OA progression^159,160^. However, in a study of the relationship between lipid and lipoprotein levels and OA in the MOST cohort, 337 symptomatic OA patients and 283 radiographic OA patients, of whom 55% were female, were included. The researchers found that the levels of total cholesterol, LDL and HDL in the serum of OA patients were not significantly correlated with joint cartilage loss, synovial inflammation or knee joint pain^161^. More basic and clinical investigations are needed to determine the role of lipoproteins in metabolic OA.

Lipoproteins are the protein component of plasma lipoproteins and are mainly divided into five categories: A, B, C, D and E. Lipoproteins are proteins that can bind and transport lipids to various tissues in the body for metabolism and utilization.

ApoA-1, the main component of HDL, plays an important role in lipoprotein clearance and cholesterol efflux in chondrocytes^154^. In a study in which 184 OA patients undergoing knee joint surgery and 180 healthy volunteers were recruited, researchers found that the expression levels of ApoA-1 in the synovial fluid (SF) of OA patients were negatively correlated with the severity of knee OA cartilage damage, radiographic severity, and severity of OA symptoms^162^. However, in a surgically induced rabbit KOA model, researchers detected a downregulation of ApoA-1 protein levels in the SF after ultrasound treatment^163^. Further exploration is needed to determine whether ApoA-1 can serve as a reliable marker for OA diagnosis.

ApoB is a basic structural component of CM, VLDL, IDL and LDL. Studies have found significant differences in ApoB levels between OA patients and healthy individuals, with higher levels in OA patients^164^. However, a bidirectional Mendelian randomization study found that elevated ApoB levels were negatively correlated with the risk of knee and hip OA^165^. Therefore, further research is needed to determine whether ApoB can serve as a molecular target for OA treatment.

ApoD is a secreted glycosylated protein and is an atypical lipoprotein that can bind to several small molecules, including arachidonic acid, steroids and cholesterol, and has important functions, such as antioxidation, anti-inflammation and anti-stress functions^166–168^. Qin et al. identified potential biomarkers for OA using weighted gene coexpression network analysis (WGCNA) and confirmed that ApoD was the only gene that was downregulated as a hub gene in multiple tissues^169^. The researchers collected serum samples from 113 KOA patients and 97 healthy controls for ELISA test and found that ApoD levels were significantly lower in KOA patients than in the control individuals, suggesting that serum ApoD levels may be associated with the severity of OA in OA patients^170^.

ApoE is an important component of plasma lipoproteins that primarily transports triglycerides and cholesterol to peripheral tissues. Farnaghi et al. generated an OA model in *ApoE*-deficient mice by feeding the mice a high-cholesterol diet for 4 weeks and found that the mice had increased osteophyte formation, aggravated cartilage degradation and more severe OA pathological symptoms^171^. In another study, researchers fed *APOE*-deficient mice with a high-cholesterol diet and found that the synovial membrane thickness increased in these mice^172^. These studies suggest that ApoE may play an important role in the occurrence and progression of metabolic OA.

ApoC is the main lipoprotein carrier of VLDL and an important regulator of lipoprotein metabolism, but there are currently almost no reports on the relationship between ApoC and OA.

### The impact of other lipid metabolism-related proteins on OA

Leptin is a hormone secreted by adipose tissue, and its serum levels are positively correlated with the size of animal fat tissue. Leptin binds to its receptor and induces cellular responses through the JAK-STAT, PI3K, AMPK and MAPK signaling pathways^173^. Studies have found that leptin has a catabolic effect on cartilage metabolism. In a study using conditioned medium from a patellar fat pad (containing leptin) derived from OA patients to treat chondrocytes, leptin significantly induced collagen release and MMP expression in chondrocytes and activated signaling pathways such as JAK-STAT^174^. Bao et al. injected recombinant rat leptin (100 μg) into rat knee joints and found that leptin significantly increased the expression levels of MMP2, MMP9, tissue protease D, and *Col-2* mRNAs and proteins and observed a decrease in proteoglycans in joint cartilage^175^. When leptin was used alone or in combination with IL-1β, it upregulated MMP production in human OA chondrocytes through signaling pathways, such as NF-κB and MAPK, leading to protein degradation of cartilage ECM^176^. In a study of 163 elderly individuals, researchers found that serum leptin levels were negatively correlated with the thickness of joint cartilage, suggesting that leptin may play an important role in changes in cartilage thickness^177^. These results suggest that leptin plays a catabolic role in cartilage metabolism and may be a detrimental factor in the pathological development of OA. However, in another study, researchers observed a significant increase in proteoglycan synthesis in all cartilage regions of the rat tibial plateau after injection of exogenous leptin (30 μg), indicating a protective effect of leptin on cartilage degradation, and these discrepancies may be dose-related^178^. Currently, the role of leptin in OA remains unclear, and further investigations are still needed.

Adiponectin, the most abundant adipokine in human plasma, is primarily secreted by white adipose tissue and plays a crucial role in regulating appetite and metabolism. Adiponectin exerts its biological effects through AdipoR1 and AdipoR2 receptors, which are expressed in various tissues, including the liver, articular cartilage, bone and synovium^179–181^. However, these two receptors have distinct functions in the body, with AdipoR1 mainly associated with AMPK signaling pathway activation, while AdipoR2 is linked to PPAR-α signaling pathway activation^182^.

In a study containing 12 patients undergoing knee replacement surgery for OA, researchers found that AdipoR1 and AdipoR2 expression levels were significantly higher in the OA cartilage lesion areas than in the non-lesion areas^180^. Moreover, the growth rate of AdipoR1-positive chondrocytes was significantly higher than that of AdipoR2-positive chondrocytes, suggesting that changes in AdipoR1 expression may better reflect the catabolic metabolism status of cartilage than AdipoR2. Adiponectin may accelerate the degradation of OA cartilage ECM through the activation of the JNK signaling pathway^180^.

**Fig. 4:**
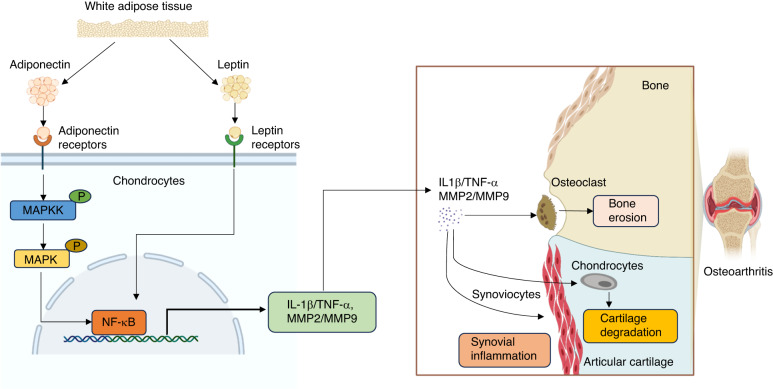
The mechanisms of leptin and adiponectin in osteoarthritis. The adiponectin and leptin secreted by white adipose tissue bind to the corresponding receptors, activate MAPK and NF-κB pathways, and promote the expression of IL-1β, TNF-α, MMP2 and MMP9. Proinflammatory cytokines and matrix metalloproteinases act on articular cartilage, subchondral bone, synovial membrane and other areas, aggravating the phenotype of osteoarthritis. MAPK mitogen-activated protein kinase, NF-κB nuclear factor-k-gene binding, IL-1β interleukin-1β, TNF-α tumor necrosis factor-α, MMP2 matrix metalloproteinases 2, MMP9 matrix metalloproteinases 9

Plasma adiponectin levels and adiponectin release from cartilage were found to be higher in patients with severe OA (Ahlbäck grades 4 and 5) than in those with mild OA (Ahlbäck grades 1 to 3). Adiponectin may activate the MAPK signaling pathway, leading to increased release of inflammatory cytokines and MMP expression in chondrocytes, thereby promoting the destruction of articular cartilage and heightening OA symptoms^183^. Similarly, in another study of OA patients undergoing total knee replacement surgery, adiponectin in knee synovial fluid significantly inhibited the aggregation of glycosaminoglycans in cartilage, suggesting that synovial adiponectin plays a positive role in cartilage damage^184^. Based on the current understanding of the relationship between adiponectin and OA, the development of pathway inhibitors related to adiponectin may be a promising avenue for the treatment of metabolic OA (Fig. 4).

### Lipid metabolism and OA treatment

Treatment of OA remains a challenging issue, with most therapeutic approaches aiming to alleviate pain, improve or restore joint function, enhance patient quality of life, delay disease progression and correct deformities. The treatment of OA requires a combination of pharmacological and nonpharmacological interventions, with surgery being necessary for severe cases in the advanced stages of the disease.

Nonpharmacological interventions include exercise and dietary management. Studies have shown that OA can be treated through nonpharmacological approaches such as aerobic and strength exercises. Regular moderate exercise can alleviate pain, improve physical function and significantly slow disease progression, making it an important component for early intervention of OA^185–189^. Overweight or obese patients with OA can achieve their ideal body weight by combining dietary adjustments with exercise, thereby reducing the burden on their joints and improving their clinical symptoms^190,191^. Overweight or obese patients with OA often have lipid metabolic disorders, and weight loss can help slow the progression of metabolic OA.

Pharmacological interventions include the use of nonsteroidal anti-inflammatory drugs (NSAIDs) either locally or systemically, which are commonly used to alleviate mild to moderate pain in OA patients^186,192^. Intra-articular injection of corticosteroids can provide short-term pain relief in OA patients. A clinical trial showed that physical therapy or corticosteroid injection had similar efficacy in the short term, but physical therapy had better long-term effects, and long-term intra-articular injection may cause some joint damage^185,193,194^. For late-stage OA patients whose pain cannot be relieved by other treatments, joint replacement surgery is recommended, which can effectively alleviate pain and improve the patient’s quality of life^195^. For the treatment of metabolic OA, targeting the pathogenesis and correcting lipid metabolism disorders may be an ideal approach.

Lipid-lowering drugs are a class of medications that can reduce plasma triglycerides or lower plasma cholesterol. They include statins, cholesterol absorption inhibitors, fibrates, PCSK9 inhibitors, niacin, bile acid sequestrants and n-3 PUFA. By targeting different types of lipid metabolic disorders, the use of different lipid-lowering drugs is a new treatment approach for metabolic OA.

Statins are competitive inhibitors of 3-hydroxy-3-methylglutaryl-coenzyme A (HMG-CoA) reductase, which can effectively lower serum cholesterol levels and are widely used to treat hypercholesterolemia^196,197^. Members of the statin class include atorvastatin, fluvastatin, lovastatin, pitavastatin, pravastatin, rosuvastatin and simvastatin^197,198^. Studies have shown that statins can delay or inhibit the progression of OA in in vitro cultured OA chondrocytes^199–203^ and experimental OA animal models in vivo^204–207^. In a clinical study comparing the progression of OA over 6.5 years between statin users and nonusers, researchers found that use of statin significantly slowed the overall progression of knee OA^208^. Another clinical follow-up study found that use of statins reduced the risk of joint space narrowing in patients with KOA compared with that of non-statin users^209^. This may be related to the anti-inflammatory and antioxidant functions of statins.

Cholesterol absorption inhibitors mainly reduce the absorption of cholesterol in the intestine and are represented by ezetimibe, which can lower low-density lipoprotein cholesterol and, when used in combination with statins, can further lower low-density lipoprotein cholesterol levels^210,211^. Although ezetimibe can significantly lower cholesterol and have therapeutic effects in other diseases, it can only lower serum cholesterol in OA and has no therapeutic effect on OA^210,212^. Niacin is converted to nicotinamide in the human body, which is a component of coenzyme I and coenzyme II, involved in lipid metabolism in the body. Currently, niacin has not been applied in the treatment of OA. Bile acid sequestration promotes the excretion of cholesterol by inhibiting the reabsorption of cholesterol-rich bile acids. However, due to their many adverse effects, they are now rarely used clinically and have not been used to treat OA.

Fibrates include fenofibrate, bezafibrate and gemfibrozil. Currently, fenofibrate is the only fibrate that is associated with OA. Fenofibrate is a peroxisome proliferator-activated receptor alpha (PPARα) agonist that can lower triglyceride levels in the body. PPARα can regulate the uptake and metabolism of fatty acids, as well as exert anti-inflammatory effects^213,214^. Studies have found that PPARα is downregulated in the blood and cartilage of surgically induced OA mouse models and KOA patients, indicating that *PPARα* deficiency may be an intrinsic factor leading to the development of OA, and PPARα agonists can prevent cartilage degradation^144^. Researchers have found that the addition of PPARα agonists to the infrapatellar fat pad (IPFP) of OA patients can effectively inhibit the production of cytokines, such as IL-6, IL-8, MCP1 and IL-4, induced by IL-1β in IPFP^215–217^. Patients with HOA showed improvement in pain, hand function, systemic inflammation and lipid status after 4 weeks of treatment with fenofibrate^218^. However, other studies have found that fenofibrate has no inhibitory effect on cartilage injury in the STR/Ort spontaneous OA mouse model^219^. Currently, the use of fibrates in the treatment of OA is still unclear and requires more basic and clinical investigations for further clarification.

PCSK9 inhibitors have potent cholesterol-lowering effects by preventing LDL receptor degradation and reducing LDL-C by 50%–70%. However, PCSK9 inhibitors are rarely used alone for lipid-lowering therapy but rather in combination with other lipid-lowering drugs for efficient lipid-lowering therapy. In a study inducing KOA in APOE∗3Leiden. CETP mice fed a high-fat diet, researchers found that cholesterol-lowering therapy with a combination of atorvastatin and PCSK9 inhibitors did not inhibit the progression of cartilage degradation in mice^220^. This suggests that while PCSK9 inhibitors can be used to treat lipid metabolic disorders, their use in the treatment of metabolic OA is still debatable.

N-3 PUFA is a type of fatty acid that cannot be synthesized by the human body. The main types of n-3 PUFA are alpha-linolenic acid, eicosapentaenoic acid (EPA) and docosahexaenoic acid (DHA), which can promote the reduction of triglycerides. In IL-1β-induced chondrocyte and synovial cell inflammation models, the addition of n-3 PUFAs can reduce the expression of inflammatory and degradation markers in chondrocytes and synovial cells^93,221^. Adler et al. found that n-3 PUFA intervention in IL-induced canine chondrocytes significantly decreased iNOS expression and NO production compared to the control chondrocytes^95^. Additionally, in human chondrocytes treated with n-3 PUFAs, the expression levels of MMP13 and PGE2 were reduced^222,223^. Therefore, the current findings suggest that n-3 PUFAs have a positive effect on chondrocytes in treating inflammation. Researchers have injected sustained-release EPA into surgically induced KOA mice and observed that it can effectively prevent the progression of KOA^224^. In a previous RCT, consuming krill oil improved pain, stiffness and functional movement in patients with mild to moderate KOA^225^. However, in another RCT, supplementing n-3 PUFAs did not alleviate knee pain, stiffness, or functional movement^226^. Although a large body of evidence suggests that n-3 PUFAs may have a role in reducing low-grade inflammation related to OA and slowing cartilage degradation, more clinical studies are needed to further clarify the function of n-3 PUFAs. Metabolic OA has been found to be associated with metabolic syndrome because they share common pathogenic factors^23^. In the future, lipid-lowering drugs combined with nonsteroidal anti-inflammatory drugs have great potential for the treatment of metabolic OA. The studies related to lipid-lowering drugs in the treatment of OA and their efficacy in OA treatment are summarized in Table 3.

**Table 3:** Lipid-lowering drugs in the treatment of OA

Lipid Lowering Drug	Drug function	Potential role in OA	Basic research	Clinical trial
Statins	TC↓ LDL↓ HDL↑	Protective effect	1. Atorvastatin may prevent the damage of the cartilage^200^. 2. Cindine promotes the repair of damaged chondrocytes^201^. 3. Pravastatin reduces the expression of MMP, promoting OA chondrocytes cholesterol efflux and protecting the chondrocytes matrix^202^. 4. Simvastatin reduces IL-1β, MMP-3, and leptin expression^203^. 5. Simvastatin delays OA progression^204^. 6. Fluvastatin attenuates the degradation of cartilage in OA^206^. 7. Lovastatin inhibits apoptosis of rabbit chondrocytes in inflammatory environments^207^.	1. Statins can significantly delay disease progression in patients with KOA^208^. 2. Statins may reduce the risk of narrowing of the joint space in people with KOA^209^.
Ezetimibe	LDL↓	No effect	Ezetimibe has no effect on inhibiting the development of OA^210,212^.	NA
Fibrates	TG↓ HDL↑	Protective effect	PPARα agonists can prevent cartilage degradation^144^.	1. PPARα agonists prevent cartilage degradation^144^. 2. PPARα agonists downregulates the production of inflammatory factors in the IPFP in patients with OA^215–217^. 3. Fenofibrate reduces pain, systemic inflammation and lipids in patients with HOA^218^.
		No effect	Fenofibrate has no inhibitory effect on the development of cartilage injury in mouse models of OA^219^.	NA
PCSK9 Inhibitors	LDL↓	No effect	PCSK9 inhibitors did not attenuate cartilage degradation in OA mice^220^.	NA
Omega-3 fatty acids	TG ↓	Protective effect	1. N-3 PUFA can reduce the expression of inflammatory factors and markers of cartilage degradation^93,221^. 2. N-3 PUFA reduces the production of iNOS and NO^95^. 3. N-3 PUFA can reduce the expression of MMP13 and PGE2^222,223^. 4. N-3 PUFA can mitigate the progression of KOA^224^.	N-3 PUFA improves pain, stiffness, and motor function in patients^225^.
		No effect	NA	N-3 PUFA cannot alleviate knee pain, stiffness and functional movement^226^.

## Perspective

Current research on lipid metabolic disorders in OA provides a basis for further exploring the pathogenesis and prognosis of this disease. A large number of studies have confirmed that while age and body weight are related to OA, lipid metabolism disorders are also involved in the progression of OA. Related in vitro and in vivo experiments have been conducted, and molecules important for metabolism, such as LXR^120,130,131^, PPARα^145^ and RORα^153,154^, have been found to play important roles in OA development. Additionally, certain lipid-lowering medications in clinical settings have been found to alleviate OA progression in recent clinical studies^144,208,209^. A combination of lipid-lowering drugs and anti-inflammatory drugs may be more beneficial for OA patients than anti-inflammatory drugs alone. However, existing data are still limited, a common mechanism for lipid metabolism disorders in OA has not been definitively revealed, and some inconsistent results have been reported. Therefore, more evidence is needed to clearly define the pathological mechanisms of lipid metabolism disorders and their contribution to OA development. Controlling body weight and a balanced diet to maintain the homeostasis of lipid metabolism can play a positive role in maintaining joint health. Finding effective drugs to target metabolic disorders will require further in-depth research in the future.
